# Targeted Molecular Screening of *Leishmania infantum* in *Sergentomyia minuta* Populations from Southern Italy

**DOI:** 10.3390/ijms27177741

**Published:** 2026-08-29

**Authors:** Desiree Brancato, Agnese Messina, Oscar Lisi, Francesca Bruno, Elvira Coniglio, Stefania Vadalà, Anna Maria Pappalardo, Venera Ferrito, Salvatore Saccone, Concetta Federico

**Affiliations:** 1 Department of Biological, Geological and Environmental Sciences, University of Catania, 95124 Catania, Italy; desiree.brancato@phd.unict.it (D.B.); agnese.messina@studium.unict.it (A.M.); oscar.lisi@unict.it (O.L.); francesca.bruno@unikore.it (F.B.); elvira.coniglio@phd.unict.it (E.C.); stefania.vadala@studium.unict.it (S.V.); pappalam@unict.it (A.M.P.); vferrito@unict.it (V.F.); concetta.federico@unict.it (C.F.); 2 Department of Medicine and Surgery, Kore University of Enna, 94100 Enna, Italy

**Keywords:** *Sergentomyia minuta*, *Leishmania* genetic diversity, ITS1, SSU rRNA, mitochondrial cytochrome b, RFLP, blood-meal analysis, vector ecology

## Abstract

*Sergentomyia minuta* is a widespread Mediterranean sand fly traditionally regarded as herpetophilic and of limited relevance for human leishmaniasis. Occasional detection of mammalian *Leishmania* DNA in *Sergentomyia* species has challenged this assumption. This study aimed to investigate the abundance, host-feeding behaviour, and molecular detection of *Leishmania infantum* DNA in *S. minuta* in peri-urban areas of southern Italy. Sand flies were collected using sticky traps at three sites in the Catania area (Italy). Unengorged females were screened for *Leishmania* spp. DNA using ITS1 and SSU rRNA markers. ITS1 PCR-RFLP was used exclusively to discriminate *L. infantum*-compatible from non-*L. infantum* molecular profiles. Engorged females were analysed for blood-meal origin using mitochondrial cytochrome b markers (CytB1 and CytB2) and a nuclear human-specific transthyretin (*TTR*) marker through PCR-RFLP and multiplex PCR. A total of 1361 *S. minuta* specimens were collected, with a strongly female-biased sex ratio. *Leishmania* spp. DNA was detected in a substantial proportion of pooled samples. ITS1 PCR-RFLP profiles revealed heterogeneous molecular patterns that consistently excluded *L. infantum* and were compatible with non-*L. infantum* trypanosomatid DNA. Blood-meal analyses suggested feeding on multiple vertebrate hosts, including occasional detection of human mitochondrial DNA, whereas the nuclear *TTR* marker was consistently negative, likely reflecting DNA degradation during blood digestion. *S. minuta* populations from southern Italy display high abundance, ecological plasticity, and a strong association with peri-urban habitats. Our findings refine, rather than overturn, the current understanding of the epidemiological role of *S. minuta* in Mediterranean ecosystems. Multilocus molecular screening revealed heterogeneous non-*L. infantum* trypanosomatid DNA patterns, while no evidence of *Leishmania infantum* was detected. Occasional human blood meals were detected, but the epidemiological relevance of *S. minuta* for human leishmaniasis remains unproven. These findings highlight the importance of integrated entomological and molecular approaches for careful interpretation of vector–parasite–host relationships Mediterranean ecosystems.

## 1. Introduction

Leishmaniases are a group of endemic parasitoses occurring in tropical, subtropical and temperate regions, characterized by diverse clinical manifestations and tissue tropisms. These diseases are reported in approximately 90 countries worldwide and account for an estimated 600,000–1,000,000 new cases of cutaneous leishmaniasis and about 14,600 deaths annually, according to the most recent global estimates [1,2,3]. Leishmaniases are caused by flagellated protozoan parasites of the genus *Leishmania* (Trypanosomatidae), which induce cutaneous (CL), mucocutaneous (MCL) and visceral (VL) leishmaniasis in humans and other mammals [4,5,6].

In the Mediterranean basin, three *Leishmania* species are recognized: *L. major* Yakimoff & Schokhor, 1914, *L. tropica* Wright, 1903, and *L. infantum* Nicolle, 1908. Of these, *L. infantum* is the only species consistently reported in Italy and represents the main etiological agent of human and canine leishmaniasis [7,8].

Recent molecular surveys conducted outside Europe have also reported the detection of *Leishmania* DNA in wild-caught *Sergentomyia* species in different ecological contexts, further highlighting the need for geographically contextualized field studies and cautious interpretation of molecular findings [9,10].

The main vectors of these anthropozoonoses are dipterans known as sand flies, belonging to the genus *Phlebotomus* Rondani & Bertè, 1840 in the Afro-Eurasia, and *Lutzomyia* França, 1924 in the Americas. These insects spread the disease in endemic areas, typically warm environments with moderate-to-high humidity. In Italy, seven different species of *Phlebotomus* and one species of *Sergentomyia*, namely *S. minuta* Rondani, 1843, have been described. This latter species shows a significant relative abundance throughout the country [11], particularly in eastern Sicily, where its presence reaches 70% in some sites compared to 30% of *Phlebotomus* spp. [12], and where leishmaniasis seroprevalence in both dogs and humans reached 332 CL cases and 135 VL cases reported between 2013 and 2021 [13]. Recent studies have further confirmed the persistence of *S. minuta* in urban sites of Sicily and other southern European regions, highlighting potential occasional contact with humans [14,15].

For a long time, only *Phlebotomus* sand flies were considered responsible for the transmission of leishmaniasis in humans and dogs across the Mediterranean basin; in contrast, *Sergentomyia* was thought to take blood meals exclusively from cold-blooded animals such as lizards and geckos. Indeed, *S. minuta* is a known vector of *Leishmania (Sauroleishmania) tarentolae* Ranque, 1973, the agent of reptile leishmaniasis [16]. Nevertheless, several studies suggest that *Sergentomyia* may also play a role in the transmission of mammalian leishmaniasis, with potential public health impact [17].

Several surveys have reported the detection of *Leishmania* DNA in *Sergentomyia* spp. and the identification of reptilian and mammalian blood meals in their midgut, demonstrating their ability to feed on the blood of both reptiles and mammals. For instance, *L. infantum* was detected by molecular methods in *S. dubia*, *S. magna* and *S. schwetzi* in Senegal [18]. *S. minuta* was found positive for *L. major* DNA in Tunisia and Portugal [19,20], and later for *L. infantum* in Portugal [21] and Italy [16]. More recently, *L. infantum* has been detected again in *S. minuta* from southern Italy [14], while ecological modelling confirmed unexpected host–parasite interactions between *S. minuta* and *L. infantum* in the Italian context [15]. Furthermore, laboratory experiments demonstrated that *S. minuta*—though predominantly reptile-oriented—can occasionally feed on human blood [22,23]. Despite these findings, vector competence in nature remains unproven, as emphasized by recent negative results in related species [10] and field studies failing to confirm transmission [24].

According to Maia and Depaquit [25], an insect can be considered a competent vector only if it is both capable of harbouring and transmitting the pathogen, and of feeding on blood of the host in which transmission occurs. Therefore, in addition to detecting *Leishmania* DNA in *Sergentomyia*, the identification of human [18] or other mammalian blood meals [26,27] is required to support its role.

Leishmaniasis represents a paradigm of the One Health concept, as its epidemiology is shaped by interactions among humans, domestic and wild animal reservoirs, sand fly vectors and environmental factors. Understanding the feeding behaviour and host preferences of *Sergentomyia* species is thus essential not only to evaluate their potential vector competence, but also for designing integrated surveillance and control measures [28,29]. In this context, particular attention has recently been drawn to *S. minuta* following the molecular detection of *L. infantum* in this species in southern Italy [14,16]. These findings raised the question of whether *S. minuta* might sporadically participate in the transmission cycle of *L. infantum* in Mediterranean endemic areas.

The present study investigates the potential role of *S. minuta* in leishmaniasis transmission in Sicily, a region where 25 symptomatic VL cases were reported in 2005 and approximately 47% of the population lives in areas at risk [30], with canine seroprevalence reaching up to 60% [31].

Specifically, the primary aim of this work was to verify the presence of *L. infantum* in field-collected *S. minuta* specimens from Sicily, in order to assess whether this species could be involved in the local transmission of visceral leishmaniasis. While advanced multilocus and amplicon-based next-generation sequencing approaches have recently been proposed for the simultaneous characterization of sand flies and associated trypanosomatids [32], the present study was not designed to systematically investigate the full spectrum of *Leishmania* or other parasites potentially harboured by *S. minuta*, but rather to assess the presence or absence of *L. infantum* in this sand fly species in a highly endemic Mediterranean setting. By integrating entomological surveys with multi-locus molecular analyses, including parasite detection and blood-meal identification, our results contribute to a more accurate assessment of the epidemiological significance of *S. minuta* in Sicily and provide further evidence relevant to the ongoing debate on its role as a potential vector of *L. infantum*.

## 2. Results

### 2.1. Entomological Survey and Population Structure of S. minuta

A total of 1361 *S. minuta* specimens were collected during the study period. Morphological identification using dichotomous keys allowed species-level identification and sex determination, revealing 1041 females and 320 males, with an overall male-to-female ratio of 0.31.

Sex ratio was evaluated for each sampling station. In all sites, females were consistently more abundant than males, confirming a marked female predominance in the sampled populations (Table 1).

Since only females have epidemiological relevance, abundance comparisons among sampling sites were performed exclusively on female specimens. Abundance was calculated as the number of females collected at each station divided by the total number of traps used. Site 3 showed the highest abundance, accounting for 63.82% of all females collected, followed by Site 1 (23.51%) and Site 2 (12.67%) (Table 2). Notably, site S3 represents the most anthropized area and was the only site where resting places consisted of drainpipes rather than wall crevices. These environmental conditions may have contributed to the markedly higher abundance observed at this station.

Regarding the physiological status of females, blood-fed individuals were detected only at sites S1 and S3, where 5 (1.6%) and 27 (4.9%) specimens, respectively, contained a recent blood meal; no engorged females were recorded at S2. Ovarian development was observed at all sites, with six females at S1 (1.9%), one at S2 (0.9%), and two at S3 (0.4%). These findings indicate that, although female abundance was consistently high, only a small fraction of the population showed evidence of recent blood feeding or egg development (Table 3).

### 2.2. Detection and Molecular Identification of Leishmania spp. in S. minuta

**Figure 1:**
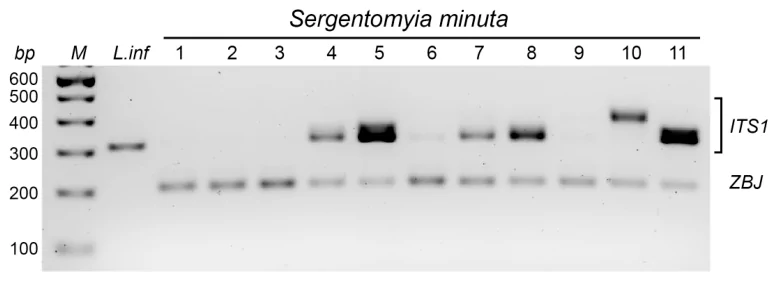
DNA extracted from pools of *S. minuta* amplified using primers specific for the ITS1 region. Each lane (1–11) corresponds to a pool of five *S. minuta* specimens. M: 100 bp molecular weight marker (bp sizes indicated on the left). *L. inf*: positive control containing *L. infantum* DNA, showing the expected ~320 bp band. ITS1 amplicons compatible with *Leishmania* spp. observed in six pools (lanes 4, 5, 7, 8, 10, and 11). Lane 10 shows an atypical fragment of approximately 400 bp, not previously described. All pools were positive for the *ZBJ* marker (arthropod DNA), confirming the presence of *S. minuta* genomic material.

A total of 250 unengorged *S. minuta* females were screened for the presence of *Leishmania* DNA. Specimens were grouped into 50 pools of five individuals each, comprising 15 pools from S1, 10 from S2, and 25 from S3, prior to genomic DNA extraction. All pools were initially analysed using the ITS1 marker (Figure 1), resulting in an overall positivity rate of 52% for *Leishmania* spp. DNA. ITS1 positivity was observed in 7/15 pools from S1 (46.7%), 6/10 from S2 (60.0%), and 13/25 from S3 (52.0%). No statistically significant differences in ITS1 pool positivity were observed among the three sampling sites (χ^2^ = 0.427, df = 2, *p* = 0.808). Figure 1 shows a representative subset of 11 pools and does not represent the complete set of 50 pools analysed.

Among ITS1-positive pools, fragment sizes were consistent with those previously described for *Leishmania* spp. Notably, no pool showed exclusively the canonical ITS1 profile attributable to *L. infantum*. An atypical ~400 bp fragment was detected in 19% of samples, while 31% displayed a double-band profile consisting of a ~350 bp fragment and the ~400 bp fragment. The ~400 bp amplicon, not previously reported for known *Leishmania* species, suggests the presence of genetically non-*L. infantum* lineages or marked intraspecific variability.

**Figure 2:**
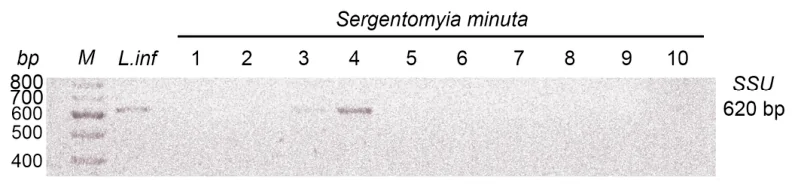
DNA extracted from individual *S. minuta* specimens (lanes 1–10) amplified using SSU-specific primers. M: 100 bp molecular weight marker (bp sizes indicated on the left). *L. inf*: positive control containing *L. infantum* DNA, showing the expected 620 bp fragment. SSU amplicons compatible with *Leishmania* spp. are visible in two samples (lanes 3 and 4).

To assess whether ITS1-negative samples contained low levels of *Leishmania* DNA, the remaining 48% of pools were analysed using the more sensitive SSU rRNA marker. This second screening identified additional positives corresponding to 8% of the previously negative pools, increasing the overall detection of *Leishmania* DNA to 56% (ITS1^+^ and/or SSU^+^) (Figure 2). The combined use of ITS1 and SSU thus increased diagnostic sensitivity and revealed genetic diversity that would not have been detected using a single-locus approach.

### 2.3. RFLP Analysis Reveals Genetic Heterogeneity and Absence of L. infantum

**Figure 3:**
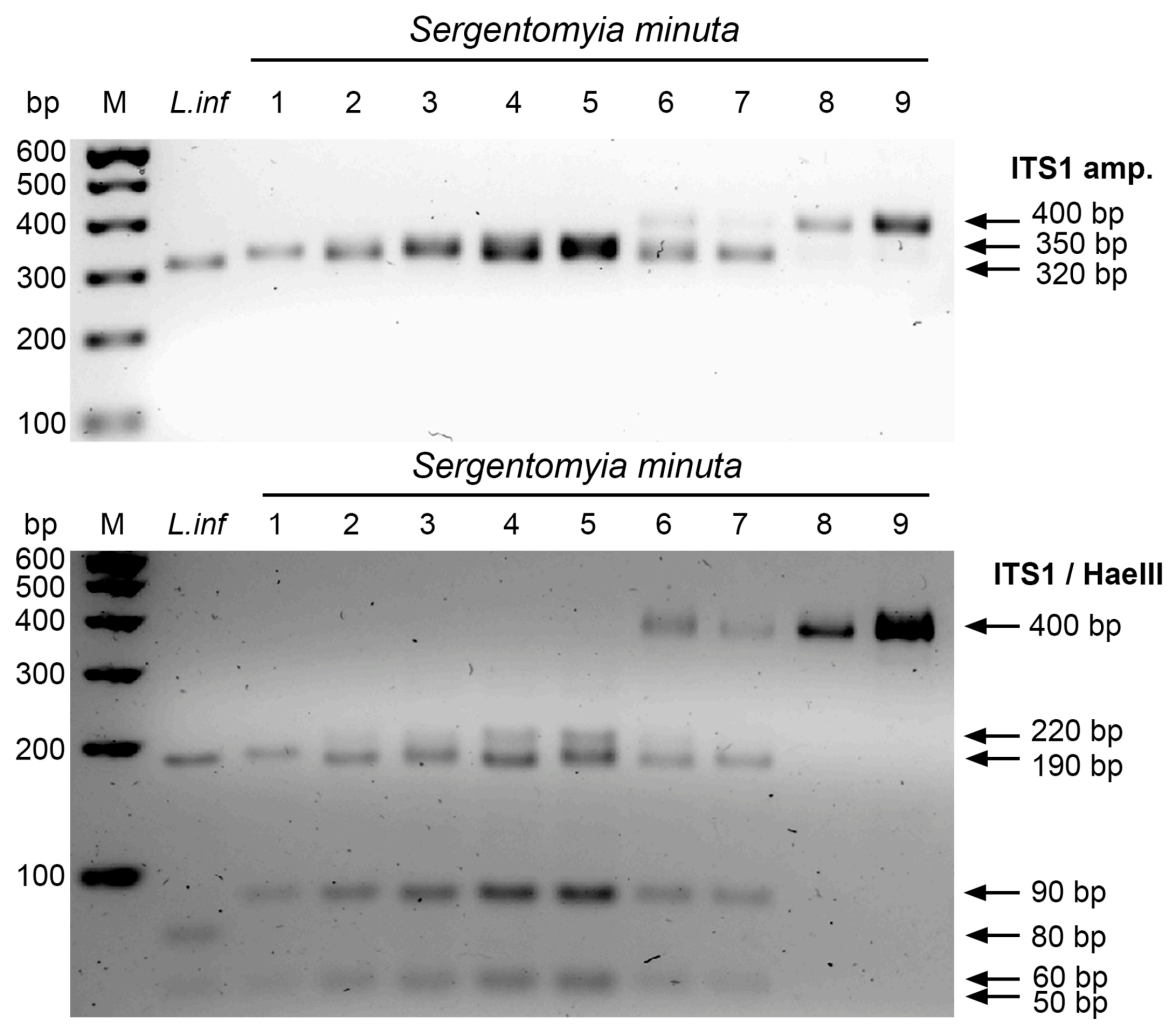
Detection and RFLP analysis of *Leishmania* spp. in *S. minuta*. Upper panel: ITS1 amplification from DNA extracted from *S. minuta*, yielding PCR products ranging from approximately 320 to 400 bp. Lower panel: HaeIII digestion of the corresponding ITS1 amplicons. DNA fragments of 320–350 bp generated restriction profiles consistent with *Leishmania* species, whereas none of the samples showed the characteristic pattern of *L. infantum*. M: 100 bp molecular weight marker (bp sizes indicated on the left). *L. inf*: positive control of *L. infantum*, showing the expected digestion products (190, 80, and 50 bp). Only non-*L. infantum*-compatible molecular profiles were detected in *S. minuta* specimens. ITS1 amplicons of approximately 400 bp remained undigested. Sizes of undigested and digested DNA fragments are indicated on the right.

The genetic diversity of *Leishmania* detected in *S. minuta* was further investigated by RFLP digestion of ITS1 PCR products using the restriction enzyme HaeIII (Figure 3). Overall, 82% of ITS1 amplicons—including those obtained after re-amplification of SSU-positive samples—were successfully digested, whereas 18% remained undigested, retaining a single band.

In samples yielding a single ~350 bp ITS1 amplicon, HaeIII digestion produced three distinct fragments consistent with restriction profiles of *Leishmania* species other than *L. infantum*. Conversely, no sample displayed the diagnostic restriction pattern characteristic of *L. infantum*, independently confirming the absence of this zoonotic species in the analysed populations. ITS1 amplicons of ~400 bp were uniformly resistant to HaeIII digestion. In samples showing a double-band pattern (350–400 bp; 31% of samples), digestion selectively affected the 350 bp fragment, while the 400 bp fragment remained intact. This differential enzymatic behaviour supports the hypothesis that the ~400 bp fragment represents a genetically non-*L.infantum* lineage compared to commonly described *Leishmania* species.

Overall, these results indicate significant genetic heterogeneity among *Leishmania* associated with *S. minuta*, with systematic exclusion of *L. infantum*.

### 2.4. Molecular Identification of Vertebrate Hosts in S. minuta Blood Meals

**Figure 4:**
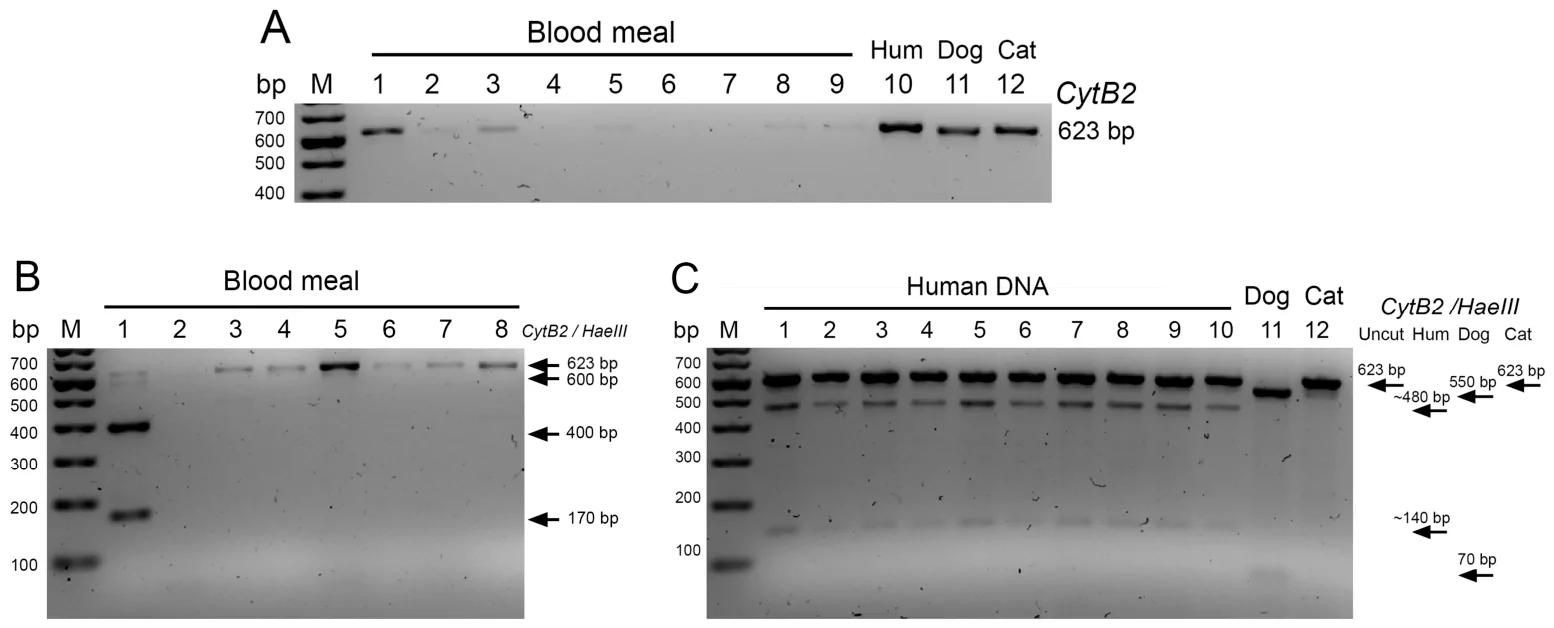
Confirmation of human blood meal by CytB2 PCR-RFLP. (**A**) CytB2 amplification (623 bp) from DNA extracted from *S. minuta* blood meals (lanes 1–9) and from three mammalian controls (human, dog, and cat). (**B**) CytB2 amplicons after digestion with HaeIII. The goat-specific restriction pattern is visible in the lane 1 sample (bands of 400 and 170 bp). In some samples (lanes 2–8), only the undigested band (~623 bp) is present. (**C**) HaeIII digestion showing the restriction profiles of human (bands of ~480 and ~140), dog (bands of 550 and 70), and cat DNA (undigested band).

To identify vertebrate hosts exploited by *S. minuta*, and specifically to assess the presence of human-derived DNA, engorged females were analysed using the mitochondrial markers CytB2 and CytB1. Blood meals were examined in 26 engorged *S. minuta* females. Amplification of the CytB2 marker was successful in 81% of specimens. RFLP analysis of CytB2 amplicons revealed distinct patterns and, in one case, fragments compatible with a mixed blood meal, including a pattern diagnostic for goat DNA (Figure 4). This observation indicates that *S. minuta* feeds on multiple mammalian hosts and may acquire mixed blood meals. No CytB2/HaeIII patterns compatible with human DNA were detected (Figure 4B,C).

**Figure 5:**
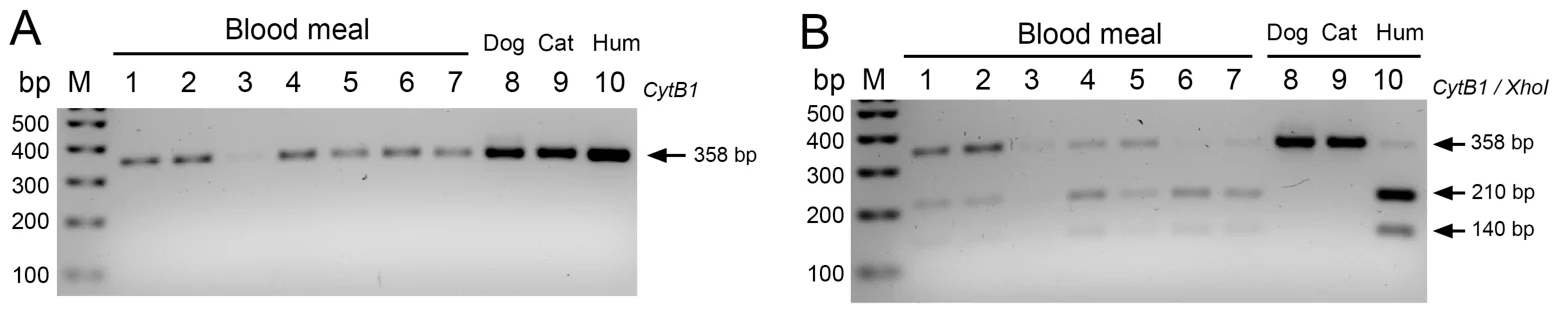
Confirmation of human blood meal by CytB1 PCR-RFLP. (**A**) CytB1 amplification (358 bp) from DNA extracted from *S. minuta* blood meals (lanes 1–7) and from three mammalian controls (human, dog, and cat). (**B**) CytB1 amplicons after digestion with XhoI. The human-specific restriction pattern is visible in the samples shown (bands of 210 and 140 bp). In some samples, an undigested band (~358 bp) is also present together with the digested fragments, suggesting mitochondrial heteroplasmy in the human DNA detected in *S. minuta*.

To confirm the presence of human DNA, a second mitochondrial marker (CytB1) was employed. CytB1 amplification was successful in 100% of samples, and digestion with XhoI produced restriction patterns diagnostic for human hosts (Figure 5). In some specimens, the presence of both digested and undigested fragments was observed, consistent with mitochondrial heteroplasmy in human DNA detected within sand fly blood meals.

Together, these results indicate that *S. minuta*, although not considered a primary anthropophilic species, is capable of feeding on mammals, including occasional feeding on humans. The combined use of CytB2 as an exploratory marker and CytB1 as a confirmatory marker increased the reliability of vertebrate host identification.

**Figure 6:**
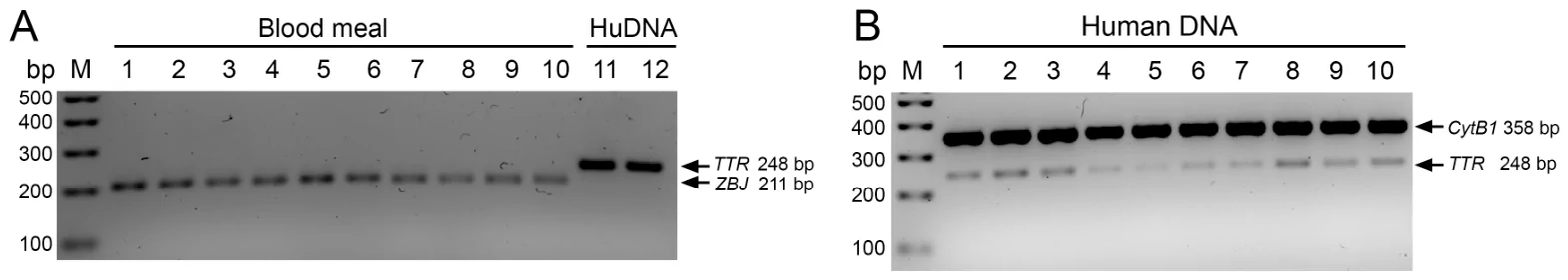
Evaluation of human nuclear DNA absence in *S. minuta* blood meals. (**A**) Multiplex PCR targeting the human-specific nuclear transthyretin gene (*TTR*) and the arthropod-specific *ZBJ* gene. Lanes 1–10 correspond to DNA extracted from *S. minuta* blood meals, positive for *ZBJ* and negative for *TTR* (*ZBJ*^+^/*TTR*^−^). Lanes 11–12 contain human control DNA, positive for *TTR* and negative for *ZBJ* (*ZBJ*^−^/*TTR*^+^). (**B**) Co-amplification of the mitochondrial marker CytB1 and the nuclear marker *TTR* from human DNA. Comparison of band intensities highlights a markedly stronger amplification of mitochondrial DNA relative to nuclear DNA.

In parallel, a multiplex PCR targeting the human nuclear gene *TTR* and the arthropod-specific gene *ZBJ* was used as a methodological control (Figure 6). Human nuclear DNA was not detectable in sand fly blood meals, whereas *ZBJ* amplification confirmed the integrity of sand fly DNA. This discrepancy between mitochondrial and nuclear DNA detection is consistent with differences in copy number and degradation dynamics and does not reflect a methodological limitation, in agreement with previous studies showing that host DNA remains detectable for a limited time depending on storage conditions [33]. Overall, these findings indicate that *S. minuta* occasionally feeds on humans, with detectable human DNA in blood meals being predominantly of mitochondrial origin.

## 3. Discussion

### 3.1. Ecology and Population Structure of S. minuta

In our survey, *S. minuta* was collected in remarkable numbers, with a total of 1041 females. This abundance is consistent with previous studies in southern Italy, where the species has been reported as well-represented in sand fly communities and occasionally reaches high densities in specific habitats [12,14,16]. The observed levels in our study are comparable or even higher than those reported in earlier entomological surveys, suggesting that *S. minuta* can thrive under diverse environmental conditions and achieve substantial population sizes.

Recent ecological modelling has further highlighted the widespread suitability of Italian habitats for *S. minuta*, confirming its ability to colonize both rural and peri-urban areas [15]. Our findings, in agreement with this literature, reinforce the view of *S. minuta* as a highly adaptable species capable of sustaining abundant populations across varied ecological contexts.

Within our study, the highest female abundance was recorded at the S3 site, the most anthropized area sampled. This suggests that the species can establish strong associations with urban environments, where artificial resting sites and abundant vertebrate hosts provide favourable conditions for sustaining dense populations.

The overall sex ratio observed (males/females = 0.31) revealed a markedly female-biased population structure. This contrasts with most entomological surveys in Italy, where sex ratios are often close to parity or male-biased [14,16]. Several factors may contribute:

- Our sampling focused on diurnal resting sites (wall holes and drainpipes), which are known to enrich for females seeking humid refugia before oviposition.
- The most anthropized site (S3) exhibited the highest abundance, suggesting that urban microhabitats amplify artificial refugia and host availability, potentially skewing the female representation [14,15,34].
- Methodological differences can bias captures: light-attraction methods typically favour males, whereas resting-site aspiration enriches for females [35,36].
- Seasonal timing may also influence sex ratios, as female cohorts dominate during peak activity [37].

Taken together, these observations suggest that the female-biased ratio reflects a genuine ecological signal amplified by site characteristics and sampling design. Epidemiologically, a surplus of females—i.e., the biting sex—within anthropized areas underlines the importance of targeted surveillance in urban/peri-urban microhabitats where *S. minuta* can sustain high-density female populations [14,16].

### 3.2. Diversity and Genetic Patterns of Leishmania in S. minuta

The primary objective of this study was to investigate whether *L. infantum* could be detected in field-collected *S. minuta* from eastern Sicily, following recent reports describing sporadic detection of this parasite in *S. minuta* from southern Italy [14,16]. Under the conditions examined, no molecular evidence of *L. infantum* was obtained. This finding is epidemiologically relevant because it indicates that, despite the high abundance of *S. minuta* in the investigated area and the endemic circulation of visceral leishmaniasis, this species does not appear to contribute to the local transmission cycle of *L. infantum*.

Nevertheless, ITS1 and SSU rRNA screening revealed the presence of trypanosomatid DNA in a substantial proportion of pools, a finding that is consistent with previous molecular surveys on *Sergentomyia* spp. in different geographic settings [18].

Notably, many ITS1-positive samples produced non-canonical amplicon sizes (~400 bp) or double-band profiles (350–400 bp), coupled with RFLP patterns diverging from reference profiles. These findings indicate the presence of non-*L. infantum* trypanosomatid DNA and highlight the limitations of ITS1 PCR-RFLP for species-level identification in complex ecological contexts, rather than supporting the existence of novel or cryptic *Leishmania* lineages. The detected DNA may correspond to reptile-associated *Leishmania* (e.g., *L. tarentolae*), other dixenous trypanosomatids, or even monoxenous genera such as *Leptomonas*, as well as potential mixed or degraded templates, as previously discussed for *Sergentomyia* spp. [10,24,38].

These results also highlight a limitation of ITS1 PCR-RFLP assays: extensive polymorphism and intraspecific variation may yield ambiguous restriction profiles, particularly in areas where multiple taxa co-circulate [39]. Consistently, the more sensitive SSU rRNA assay detected additional positives not identified by ITS1 alone, confirming that single-locus screening may underestimate parasite prevalence. Accordingly, multi-locus approaches combining markers with different levels of variability (e.g., SSU, ITS1, *hsp70*, *cytb*) are increasingly recommended [40,41,42].

The atypical ITS1 profiles observed in several samples therefore most likely reflect the presence of non-*L. infantum* trypanosomatid DNA, potentially including reptile-associated taxa such as *L. tarentolae*. Recent phylogenomic studies support the view that reptile-associated *Leishmania* lineages are more diverse than previously recognized and may occasionally be detected in mammalian hosts [22].

While ribosomal markers such as ITS1 retain sufficient variability for screening purposes [39], their use alone is insufficient to infer biological infection, transmission potential, species identity or evolutionary relationships. Recent studies increasingly advocate multilocus and amplicon-based Next-Generation Sequencing (NGS) approaches to overcome these limitations [32,40,41,42]. Accordingly, the heterogeneous ITS1 profiles detected in *S. minuta* should be interpreted as heterogeneous molecular patterns consistent with non-*L. infantum* trypanosomatid DNA, rather than evidence of distinct parasite lineages.

Overall, our findings refine the current understanding of the epidemiological role of *S. minuta* in Mediterranean ecosystems. Although no evidence supporting its involvement in the local transmission of *L. infantum* was obtained, the detection of heterogeneous non-*L. infantum* trypanosomatid DNA highlights the complexity of parasite circulation in non-classical sand fly species and underscores the need for sequence-based, multi-locus approaches to accurately characterize vector–parasite associations [43].

### 3.3. Feeding Behavior and Host Preferences of S. minuta

Analysis of blood meals via mitochondrial cytB genes (CytB2 and CytB1) revealed multiple vertebrate sources, including humans, while nuclear *TTR* assays remained negative. This discrepancy is consistent with known differences in copy number and persistence between mitochondrial and nuclear DNA, particularly in partially degraded blood [44].

Mitochondrial PCR–RFLP has long been a robust approach for sand fly blood-meal identification, discriminating multiple hosts and mixed meals [45,46]. Our results indicate occasional evidence of human-derived blood meals, but do not support frequent anthropophagy or imply epidemiological relevance, a finding supported by experimental studies demonstrating occasional human feeding despite reptilian host preference [22].

Although mitochondrial markers may increase sensitivity, the possibility of low-level contamination or mixed blood meals cannot be entirely excluded, and the results should therefore be interpreted as indicative of occasional anthropophagy rather than systematic host preference. Epidemiologically, occasional human blood feeding may increase the potential for transmission of human-infecting pathogens, though the role of *S. minuta* in leishmaniasis transmission appears limited under the studied conditions. Multi-marker approaches, potentially including NGS, improve detection of mixed or degraded blood meals and better capture opportunistic feeding behaviour.

### 3.4. Limitations

Although this study provides detailed insights into the ecology, feeding behaviour, and *Leishmania* detection patterns of *S. minuta*, several limitations should be acknowledged. First, our sampling strategy focused on resting sites and employed sticky traps, which could have biased the sex ratios in favour of females. Second, while samples were initially analysed in pools to optimize efficiency, the approach did not compromise the detection of genetic heterogeneity, as mixed profiles were subsequently resolved at the individual level. Third, partial degradation of blood-meal DNA, especially for nuclear markers, may have limited the sensitivity of vertebrate-host identification, possibly underestimating occasional anthropophagy. Fourth, ITS1 and SSU rRNA markers provide only partial resolution for species-level identification, and rare or cryptic *Leishmania* lineages might have remained undetected. Despite these limitations, the combination of multi-locus detection, RFLP analysis, and blood-meal characterization provides a robust framework for assessing the ecology and epidemiological potential of *S. minuta* populations. Finally, the study was not designed to assess vector competence, which requires experimental transmission, parasite development within the vector, and demonstrated infectivity to vertebrate hosts [25].

### 3.5. Future Perspectives

Future studies employing sequence-based and amplicon-based NGS approaches may clarify the relevance of sand flies and associated trypanosomatids [32,43]. Such methods would allow the detection of rare or cryptic parasite lineages, provide more precise host assignment for mixed or degraded blood meals, and improve understanding of vector–parasite–host interactions. Integrating genomic and ecological data will enhance surveillance strategies and risk assessment, particularly in Mediterranean regions undergoing environmental change and increased human mobility.

## 4. Materials and Methods

### 4.1. Study Area

The study was conducted in Catania, located on the eastern coast of Sicily, Italy. The area has a typical Mediterranean climate, with mild, short winters and long, hot summers, although some inland areas may exhibit more continental features. Sampling was performed between October 2018 and September 2019, divided into two collection seasons.

During the first season (2018), sand flies were collected at two sites previously described by Lisi et al. [12] as having the highest abundance of *S. minuta*. In the second season (from July 2019), an additional site in the northern urban area, not previously investigated, was included. This site comprises two closely located sub-sites; specimens from both sub-sites were pooled for analysis and are hereafter referred to as PLN1/2. A summary of all sampling stations is provided in Table 4.

Sites were selected based on environmental features, including high human population density, intense anthropogenic activity, and the presence of abundant cold-blooded fauna near uncultivated residual areas. These factors were considered potentially favourable for the establishment of suitable microhabitats for sand flies, particularly *S. minuta*.

### 4.2. Sample Collection and Identification of Sand Flies

Sand flies were collected using sticky traps, as described by Lisi et al. [12]. The sampling schedule is shown in Table 5. In each event, an equal number of traps was placed per station, although the number of recollected traps varied due to environmental factors such as rain, wind, or rodent interference. Traps were deployed at sunset and retrieved after 72 h, as commonly adopted for sand fly surveillance.

In the laboratory, insects were removed from the adhesive surfaces with a soft brush to minimize damage and preserved in 90% ethanol at room temperature. Morphological identification was performed using dichotomous keys [47]. Only *S. minuta* females were selected for further analysis. Specimens were classified as “engorged” or “unengorged” based on the presence or absence of a blood meal and stored at –20 °C. Males were excluded, as they do not feed on blood and therefore are not relevant for pathogen-transmission studies.

### 4.3. DNA Extraction and PCR Analysis

Unengorged females of *S. minuta* were grouped into pools of five individuals and subjected to genomic DNA extraction. DNA was extracted using the MagCore Compact Automated Nucleic Acid Extractor (RBC Bioscience, New Taipei City, Taiwan; Cat. No. MCA0801) in combination with the MagCore Genomic DNA Tissue Kit (RBC Bioscience, Taiwan; Cat. No. MGT-02), according to the manufacturer’s instructions.

Extracted DNA was screened for the presence of *Leishmania* spp. using two molecular targets: the internal transcribed spacer 1 (ITS1) region and the small subunit ribosomal RNA (SSU rRNA) gene. In parallel, amplification of the *ZBJ* marker was performed as an internal control to confirm the presence and integrity of arthropod DNA. The primers used and the expected amplicon sizes are reported in Table 6.

PCR amplifications were performed following the protocol described by Schönian et al. [39], using Invitrogen™ Platinum™ Taq DNA Polymerase (Invitrogen™, Cat. No. 10966018) and an Applied Biosystems Veriti Thermal Cycler (Thermo Fisher Scientific, Foster City, CA, USA). Amplification of ITS1 and *ZBJ* targets was carried out under the following conditions: initial denaturation at 94 °C for 5 min, followed by 35 cycles of denaturation at 94 °C for 45 s, annealing at 60 °C for 60 s, and extension at 72 °C for 60 s. Amplification of the SSU rRNA gene was performed with an initial denaturation at 94 °C for 5 min, followed by 38 cycles at 94 °C for 40 s, 58 °C for 60 s, and 72 °C for 100 s.

PCR products were resolved by agarose gel electrophoresis, stained with SYBR Safe DNA Gel Stain (Thermo Fisher Scientific, Foster City, CA, USA), and visualized using a Safe Imager™ 2.0 Blue-Light Transilluminator (Thermo Fisher Scientific, Foster City, CA, USA). For samples ITS1-negative, but positive at the SSU rRNA screening, ITS1 PCR products were re-amplified to increase sensitivity, as previously described [39,48]. Although referred to as nested PCR in earlier literature, this step involved a second amplification using the same ITS1 primers rather than internal nested primers.

An assay-specific limit of detection was not independently determined under the experimental conditions of the present study. However, the analytical sensitivity of the same ITS1 (LITSR/L5.8S) and SSU rDNA (R221/R332) primer systems was previously evaluated by Schönian et al. [39] using human and canine blood seeded with known numbers of *L. donovani* or *L. infantum* promastigotes. Under those validation conditions, parasite DNA corresponding to 0.2 parasite equivalents per PCR could be detected, with SSU rDNA-PCR generally showing higher sensitivity than conventional ITS1-PCR.

### 4.4. RFLP Analysis of ITS1-PCR Products

RFLP patterns were obtained using the HaeIII restriction enzyme (Thermo Fisher Scientific, Foster City, CA, USA, Cat. No. ER0151), according to Shonian et al. [39]. A total volume of 20 µL, containing 2 µL of enzyme buffer, 10 µL of unpurified PCR product, 1 µL of endonuclease and water up to the final volume, was used to carry out the digestion reaction. The reaction mixture was incubated for 16 h at 37 °C. Restriction fragments were separated on 2.5% high-resolution agarose, in 0.5× TBE buffer, and their sizes were determined by comparison with a TrackIt™ 100 bp DNA ladder (Invitrogen, Thermo Fisher Scientific, Foster City, CA, USA, Cat. No. 10488-058).

### 4.5. Blood Meal Identification and Analysis

Engorged females of *S. minuta* were processed individually and subjected to genomic DNA extraction using the same automated protocol described above. To identify the sand fly species and discriminate blood meal sources, including reptilian hosts, a rapid and reliable molecular approach based on DNA amplification followed by enzymatic digestion was applied [52]. This method generates distinct restriction patterns that allow accurate identification of *Sergentomyia* species as well as vertebrate blood sources.

To characterize the blood meal source, each sample was analysed through the amplification of two mitochondrial DNA regions of the cytochrome b (CytB) gene. The CytB2 and CytB1 regions were amplified according to Maleki-Ravasan et al. [45], with the following conditions: 95 °C for 5 min, followed by 35 cycles at 95 °C for 60 s, 58 °C for 60 s, and 72 °C for 60 s. PCR products were digested with HaeIII (CytB2) or XhoI (CytB1) enzyme, and the fragments were separated on a 2% agarose gel (Sigma-Aldrich, Sigma Aldrich, Darmstadt, Germany, Cat. No. 05066-50g).

In addition to CytB amplification, a multiplex PCR was performed to simultaneously amplify the human transthyretin (*TTR*) gene and a 211 bp fragment of the arthropod mitochondrial COI gene, using primers *ZBJ*-ArtF1c and *ZBJ*-ArtR2c [50,51,53]. This combined approach allowed confirmation of human DNA presence while verifying the integrity of sand fly DNA. PCR products and primer sequences are reported in Table 6.

The molecular workflow was specifically designed as a targeted screening strategy for *L. infantum*, rather than for comprehensive taxonomic characterization of trypanosomatids. Therefore, ITS1 PCR-RFLP was used as a screening tool to discriminate *L. infantum* from non-*L. infantum* profiles, whereas sequence-based identification of atypical amplicons was beyond the scope of the present study.

Accordingly, representative PCR-RFLP profiles were interpreted only in relation to the presence or absence of the diagnostic *L. infantum* restriction pattern, and not as evidence for species identification or taxonomic assignment of non-*L. infantum* trypanosomatids.

### 4.6. Statistical Analysis

Differences in ITS1 pool positivity among sampling sites were evaluated using Pearson’s chi-square test. Statistical significance was set at *p* < 0.05. Statistical analyses were performed using GraphPad Prism version 8.0 (GraphPad Software, San Diego, CA, USA).

## 5. Conclusions

Our findings refine, rather than overturn, the current understanding of the epidemiological role of *S. minuta* in Mediterranean ecosystems. This study demonstrates that *S. minuta* populations from the Catania area frequently harbour non-*L. infantum* trypanosomatid DNA, while no molecular evidence of *L. infantum*, the main agent of human leishmaniasis in the Mediterranean basin, was detected.

The high abundance, ecological plasticity, and female-biased population structure observed indicate that *S. minuta* is well adapted to anthropized environments. Blood meal analyses suggest occasional human feeding events, although the epidemiological relevance of this behaviour remains limited under the conditions investigated.

Taken together, our findings do not support a role of *S. minuta* in the local transmission of *L. infantum*. Rather, they indicate that this species can harbour non-*L. infantum* trypanosomatid DNA, without providing evidence of vector competence or involvement in the transmission of human leishmaniasis.

This study underscores the importance of targeted, hypothesis-driven field investigations combined with conservative molecular interpretation and provides a robust baseline for future studies employing multilocus and next-generation sequencing approaches.

## Figures and Tables

**Table 1:** Number of male and female *S. minuta* collected at the three sampling stations.

Station	Male	Female	Total	M/F Ratio
S1	152	371	523	0.41
S2	39	114	153	0.34
S3	129	556	685	0.23

**Table 2:** Percentage and abundance of *S. minuta* female specimens sampled during the study period.

Station	Female/Traps	Abundance	Percentage (%)
S1	371/107	3.47	23.51
S2	114/61	1.87	12.67
S3	556/59	9.42	63.82

**Table 3:** Number of blood-fed females and females with developed ovaries recorded at each site.

Station	Total	Blood Meal	Eggs
S1	371	5	6
S2	114	0	1
S3	556	27	2

**Table 4:** Sampling stations and geographic characteristics.

Stations	Geographic Coordinates	Altitude (m a.s.l.) ^a^
S1 (ADO)	37°30′18″ N; 015°03′39″ E	66
S2 (CHL)	37°31′15″ N; 015°05′32″ E	98
S3 (PLN1/2)	37°32′04″ N; 015°05′32″ E	135

^a^ m a.s.l.: meters above sea level.

**Table 5:** Trap deployment schedule.

Dates	Trap Number		
	S1	S2	S3
4–7 October 2018	14	6	-
7–10 October 2018	14	7	-
10–13 October 2018	17	8	-
13–16 October 2018	16	12	-
4–7 July 2019	18	9	10
31 July–3 August 2019	16	8	16
28–31 August 2019	-	5	17
16–19 September 2019	12	6	16

**Table 6:** Primers used in this study.

Primers	Nucleotide Sequence 5′–3′	Product (bp)	Refs.
LITSR: *ITS1*-For	CTGGATCATTTTCCGATG	300–350	[48]
L5.8S: *ITS1*-Rev	TGATACCACTTATCGCACTT		
R221: SSU rDNA-For	GGTTCCTTTCCTGATTTACG	603	[49]
R332: SSU rDNA-Rev	GGCCGGTAAAGGCCGAATAG		
ArtF1c: *ZBJ*-For	AGATATTGGAACWTTATATTTTATTTTTGG	211	[50]
ArtR2c: *ZBJ*-Rev	WACTAATCAATTWCCAAATCCTCC		
UNFOR403: CytB2-For	TGAGGACAAATATCA TTCTGAGG	623	[45]
UNREV1025: CytB2-Rev	GGTTGTCCTCCAA TTCATGTTA		
CytB1-For	CCATCCAACATCTCAGCATGATGAAA	358	[33]
CytB1-Rev	CCCCTCAGAATGATATTTGTCCTCA		
*TTR-For*	TCCTCCATGCGTAACTTAAT	248	[51]
*TTR-Rev*	ACTGTGCATTTCCTGGAATG		

## Data Availability

The original contributions presented in this study are included in the article. Further inquiries can be directed to the corresponding author.

## Abbreviations

The following abbreviations are used in this manuscript:

bp	base pair
CL	cutaneous leishmaniasis
CytB1	mitochondrial cytochrome b marker 1
CytB2	mitochondrial cytochrome b marker 2
ITS1	internal transcribed spacer 1
MCL	mucocutaneous leishmaniasis
NGS	Next-Generation Sequencing
PCR	polymerase chain reaction
RFLP	restriction fragment length polymorphism
SSU rRNA	small subunit ribosomal RNA
TTR	transthyretin
VL	visceral leishmaniasis
ZBJ	arthropod-specific mitochondrial COI gene marker
